# Mitotic Phosphorylation of TREX1 C Terminus Disrupts TREX1 Regulation of the Oligosaccharyltransferase Complex

**DOI:** 10.1016/j.celrep.2017.02.051

**Published:** 2017-03-14

**Authors:** Martin Kucej, Charles S. Fermaintt, Kun Yang, Ricardo A. Irizarry-Caro, Nan Yan

**Affiliations:** 1 Departments of Immunology and Microbiology, University of Texas Southwestern Medical Center, Dallas, TX 75390, USA

## Abstract

*TREX1* mutations are associated with several auto-immune and inflammatory diseases. The N-terminal DNase domain of TREX1 is important for preventing self-DNA from activating the interferon response. The C terminus of TREX1 is required for ER localization and regulation of oligosacchariyltransferase (OST) activity. Here, we show that during mitosis TREX1 is predominately phosphorylated at the C-terminal Serine-261 by Cyclin B/CDK1. TREX1 is dephosphorylated quickly at mitotic exit, likely by PP1/PP2-type serine/threonine phosphatase. Mitotic phosphorylation does not affect TREX1 DNase activity. Phosphomimetic mutations of mitotic phosphorylation sites in TREX1 disrupted the interaction with the OST subunit RPN1. RNA-seq analysis of *Trex1*^−/−^ mouse embryonic fibroblasts expressing TREX1 wild-type or phosphor-mutants revealed a glycol-gene signature that is elevated when TREX1 mitotic phosphorylation sites are disrupted. Thus, the cell-cycle-dependent post-translation modification of TREX1 regulates its interaction with OST, which may have important implications for immune disease associated with the DNase-independent function of TREX1.

## In Brief

TREX1 has a DNase-independent function in the C terminus that regulates the ER oligosaccharyltransferase (OST) activity. Kucej et al. found that TREX1 is phosphorylated during mitosis at Serine-261 in the C terminus. Mitotic phosphorylation disrupts TREX1 interaction with the OST complex without affecting its DNase activity.

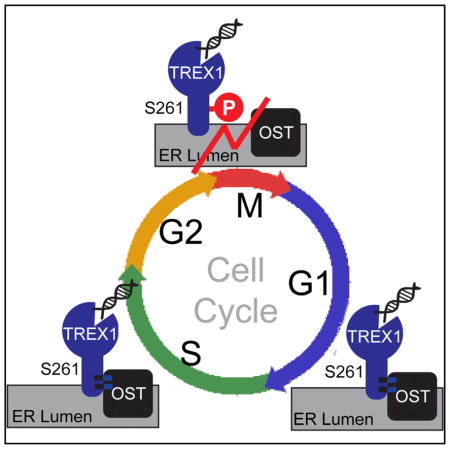

## INTRODUCTION

TREX1, also known as DNase III, is a 3′ → ′ DNA exonuclease tail-anchored at the endoplasmic reticulum (ER). Mutations in *TREX1* have been associated with a broad spectrum of autoimmune and inflammatory phenotypes, including Aicardi-Goutières syndrome (AGS), familial chilblain lupus (FCL), systemic lupus erythematosus (SLE), and retinal vasculopathy with cerebral leukodystrophy (RVCL) (Crow and Rehwinkel, 2009). Missense mutations that disrupt TREX1 DNase activity cause self-DNA to accumulate in the cytosol, which triggers the cGAS-STING innate immune sensing and type I interferon (IFN) response (Gao et al., 2015; Stetson et al., 2008). We recently showed that frameshift mutations that truncate the TREX1 C-terminal ER localization domain, without affecting DNase activity, cause dysregulation of the ER oligosaccharyltransferase (OST), leading to the release of free glycans and potentially glycosylation defects (Hasan et al., 2015). These two distinct functions of TREX1 are spatially separated into the N and C terminus of the protein. The only post-translational modification of TREX that has been described to date is the ubiquitination of TREX1 C terminus that regulates its ER localization but not the DNase activity (Orebaugh et al., 2013). No phosphorylation has been described for TREX1.

Another gene also associated with AGS is SAMHD1, a dNTPase that is at least in part regulated by cell-cycle-dependent phosphorylation (Ballana and Esté, 2015). SAMHD1 is phosphorylated on residue T592 in cycling cells and the phosphorylation is removed in non-cycling cells (White et al., 2013). Phosphorylation of SAMHD1, either by CDK1 or CDK2, is associated with lost of restrictive activity against HIV-1 replication (Ballana and Esté, 2015). Mitotic phase of the cell cycle is characterized by widespread protein phosphorylation. Many kinases are active during mitosis, three of which, CDK, PLK, and Aurora families have been most intensely studied. CDK1 is the master regulator of mitotic entry. It binds cyclin B before the entry into mitosis and the cyclin B/CDK1 complex is activated when inhibitory phosphorylation on CDK1 is removed by CDC25 phosphatase. When mitotic chromosomes are aligned and attached to spindle, CDK1 activity is turned off by proteasomal degradation of its regulatory subunit, cyclin B. It is estimated that several thousand proteins are phosphorylated by CDK1 alone (Dephoure et al., 2008). Only a fraction of this vast array of regulations have been studied. Here, we describe cell-cycle-dependent phosphorylation of TREX1 at the C terminus by mitotic cyclin B/CDK1 and demonstrate that the mitotic phosphorylation regulates the DNase-independent function of TREX1 by disrupting its interaction with the OST complex.

## RESULTS

### Cyclin B/CDK1 Phosphorylates TREX1 in Mitosis

**Figure 1:**
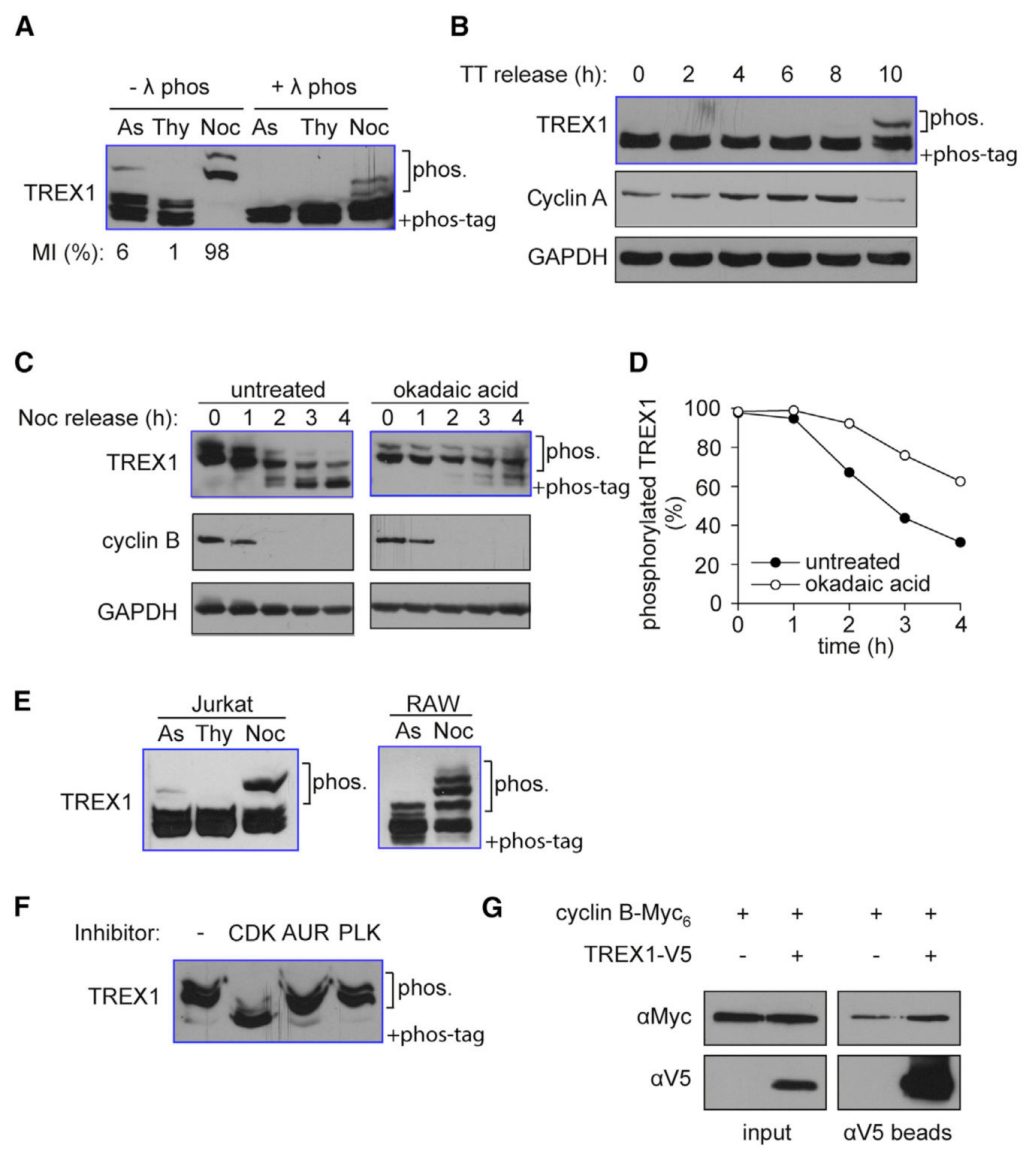
TREX1 Is Phosphorylated during Mitosis (A) HeLa cells were grown asynchronously (As), arrested with thymidine in interphase (Thy), or arrested in mitosis with nocodazole (Noc). Mitotic index (MI) measured by counting Phosphorylation of histone H3 serine 10 (p-H3S10) stained cells by fluorescent microscopy is indicated on the bottom. TREX1 phosphorylation was assayed using immunoblot of Phos-Tag PAGE (same throughout). (B) HeLa cells were arrested in G1/S transition with double thymidine treatment (TT). Cells were released and collected at indicated time points on top. Mitotic entry is indicated by degradation of cyclin A. Loading control, GAPDH. (C and D) HeLa cells were arrested in mitosis by thymidine/nocodazole treatment followed by okadaic acid (PP1/2 inhibitor) treatment, or not. Cells were released by washing out nocodazole and collected at the indicated time points. Mitotic exit is indicated by degradation of cyclin B. Loading control, GAPDH. Representative immunoblots shown in (C). Densitometry analysis of hyper-phosphorylated form of TREX1 is shown in (D). (E) Jurkat T cells or RAW267.4 mouse macrophages are treated as in (A) to enrich cells arrested at interface or mitosis. TREX1 phosphorylation is detected by immunoblot of Phos-Tag PAGE. (F) HeLa cells were arrested in mitosis by thymidine and nocodazole. Cells were treated 1 hr with RO3306 (CDK), ZM447439 (AUR), and BI2536 (PLK). (G) TREX1-V5 and cyclin B-Myc_6_ were overexpressed in 293T cells arrested in mitosis by no-codazole. Cell extracts were immunoprecipitated using anti-V5 agarose beads. Cyclin B co-immunoprecipitation was detected using anti-Myc antibody. Data are representative of at least three independent experiments.

To determine whether endogenous TREX1 protein is phosphorylated, we analyzed TREX1 protein migration in SDS-PAGE containing Phos tag, which retards migration of phosphorylated proteins (Gehrke et al., 2013). We used an antibody that specifically recognizes human TREX1 and yields a single band on immunoblots with regular SDS-PAGE (Figure S1). When we add Phos tag in the SDS-PAGE, the majority of TREX1 migrated as a double-band with a third, weaker-intensity, super-shifted band (Figure 1A). The super-shifted band disappeared after λ-phosphatase treatment, suggesting that it is a hyper-phosphorylated form of TREX1. We hypothesized that the small percentage of TREX1 phosphorylation may be due to cell-cycle regulation, since mitotic cells represent only about 6% of the population in asynchronized HeLa cells. To test this possibility, we arrested HeLa cells in G1+S phase with thymidine and in M phase with thymidine/nocodazole treatments and compared the extent of TREX1 phosphorylation. We found that TREX1 was completely phosphorylated in cells that were arrested in mitosis (Figure 1A). In comparison, another ER-localized protein STING (which is also involved in innate immune response to cytosolic DNA) did not show any mitotic-specific phosphorylation (Figure S2). To characterize the kinetics of TREX1 phosphorylation in greater detail, we synchronized HeLa cells in G1+S phase with double-thymidine treatment and measured TREX1 phosphorylation after they were released into S phase. TREX1 phosphorylation coincided with cyclin A degradation, which is an event that occurs in early mitotis (Figure 1B). To monitor de-phosphorylation kinetics, we arrested HeLa cells in mitosis with thymidine/nocodazole treatment, where the majority of TREX1 was phosphorylated and released the cells into G1 phase by washing out nocodazole. TREX1 was gradually dephosphorylated within three hours after cells exited mitosis, coinciding with the disappearance of cyclin B (Figures 1C and 1D). TREX1 dephosphorylation was inhibited by okadaic acid suggesting that PP1/PP2-type serine/threonine phosphatase is responsible for removing the phosphates (Figures 1C and 1D). The hyper-phosphorylation was also detected in human Jurkat T cells and in mouse RAW267.4 macrophages (with an antibody that recognizes mTrex1) that were partially enriched in mitosis by nocodazole treatment (Figure 1E). To see whether TREX1, in turn, regulates the cell cycle, we stained wild-type (WT) and *Trex1*^−/−^ mouse embryonic fibroblasts (MEFs) with propidium iodide (PI) and analyzed by fluorescence-activated cell sorting (FACS). We found that *Trex1*^−/−^ did not alter the cell cycle when compared to WT MEFs (Figure S3). In conclusion, TREX1 is robustly phosphorylated in mitosis and quickly dephosphorylated after mitotic exit.

There are three major types of mitotic protein kinases: cyclin-dependent kinases, Aurora kinases, and Polo-like kinases. To determine which enzyme is responsible for the mitotic phosphorylation of TREX1, we treated cells arrested by nocodazole with inhibitors of each kinase. Only the CDK1 inhibitor caused phosphorylated TREX1 to shift to the unphopshorylated form, suggesting that TREX1 mitotic phosphorylation is dependent on CDK1 (Figure 1F). CDK1 associates with cyclin A and cyclin B in mitosis. Cyclin A is degraded early during mitotic entry and is not present in Nocodazole-arrested cells in which TREX1 is robustly phosphorylated. Therefore, cyclin B is a more likely candidate for mediating TREX1 phosphorylation. To confirm this, we tested whether TREX1 interacts with cyclin B biochemically. We expressed TREX1-V5 and Cyclin B1-Myc_6_ in 293T cells enriched in mitosis by nocodazole treatment and pulled down TREX1 with anti-V5 antibody beads. Indeed, we found cyclin B1 to co-immunoprecipitate with TREX1 (Figure 1G). Together, these data suggest that cyclin B associated CDK1 kinase is responsible for TREX1 mitotic phosphorylation.

### The Major TREX1 Mitotic Phosphorylation Site Resides in the C Terminus

**Figure 2:**
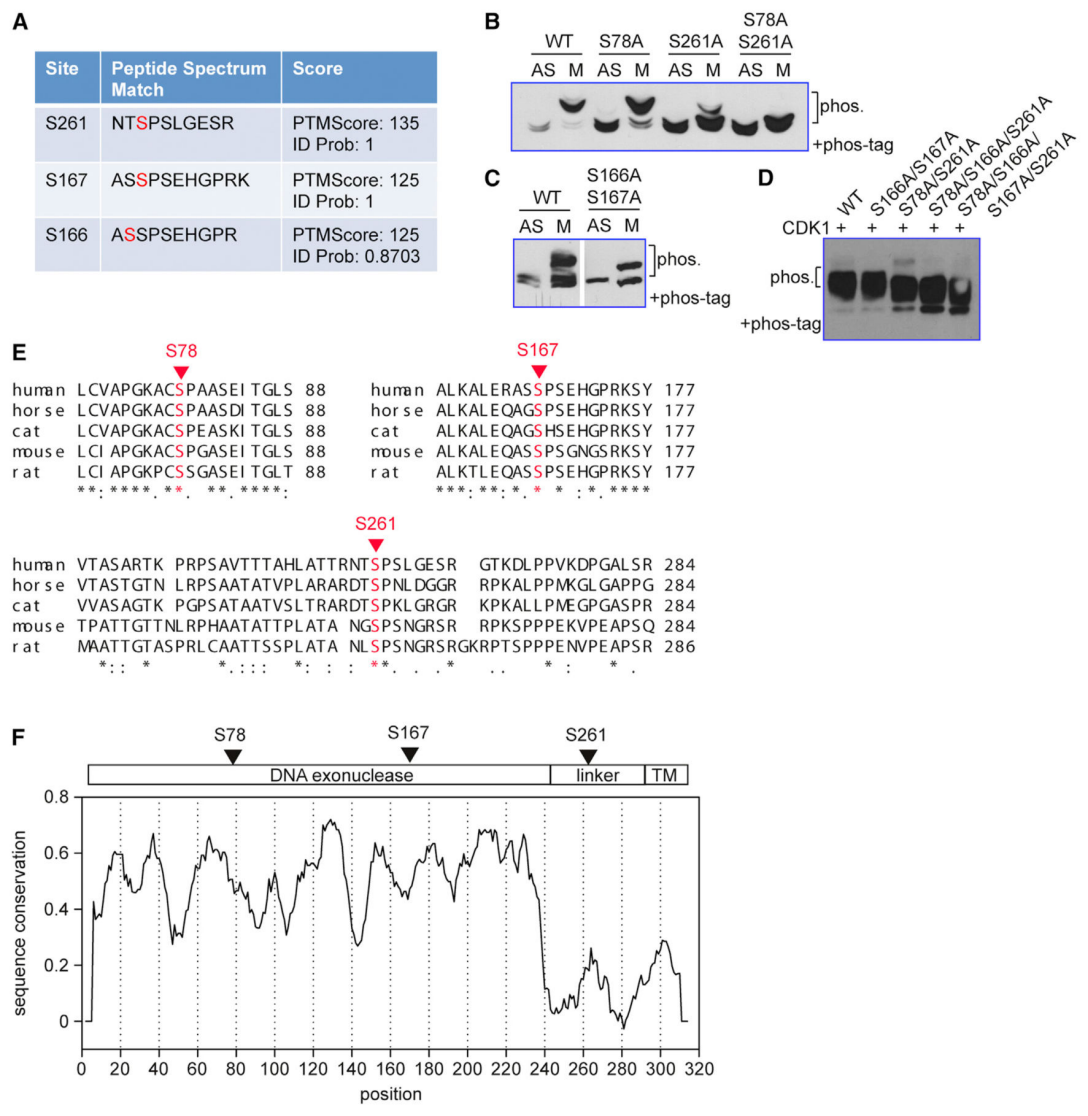
Mapping of TREX1 Phosphorylation Sites (A) Mass spectrometry findings of TREX1 post-translational modifications. Mitotic HeLa cells expressing V5-TREX1 were immunoprecipitated with anti-V5 antibody and subjected to mass spectrometry analysis. (B) Cell-cycle-dependent phosphorylation at S78/S261. WT or mutant TREX1 was expressed in HeLa cells that are asynchronized (AS) or arrested in mitosis with nocodazole (M). TREX1 phosphorylation is detected by immunoblot of Phos-Tag PAGE. (C) Cell-cycle-independent phosphorylation at S166/S167. WT or mutant TREX1 were expressed in HeLa cells and analyzed as in (B). (D) CDK1 phosphorylates mitotic phosphorylation sites on TREX1. WT or mutant TREX1 (indicated on top) were immunoprecipitated with anti-V5 antibody and incubated with recombinant CDK1 followed by immunoblot of Phos-Tag PAGE. (E) Confirmed phosphorylated sites are shown in a Clustal alignment of five mammalian TREX1 homologs. Uniprot accession numbers are human (Q9NSU2-3), horse (F6PVF5), cat (M3X2S3), mouse (Q91XB0), rat (Q5BK16). (F) Schematic diagram of TREX1 with confirmed phosphorylated sites indicated on top and sequence conservation on the bottom. The plot was generated with Plotcon, using EBLOSUM62 matrix with 10aa (amino acid) window size.

To map the phosphorylated sites, we immunoprecipitated TREX1-V5 from mitotic HeLa cells and examined the protein by mass spectrometry. About 47% of the protein (17 out of 26 serines) was covered by this analysis. This approach uncovered three phosphorylated serines, S261, S166, and S167 (Figure 2A). We mutated these serines individually to alanine or in combination as well as another residue S78 reported by a high-throughput phosphor-proteome study (Dephoure et al., 2008). Next, we transfected plasmids with these mutations into HeLa cells that were either asynchronized (AS) or arrested in mitotic (M) phase with nocodazole, and examined the level of phosphorylation by Phos tag containing SDS-PAGE (Figures 2A and 2B). WT TREX1-V5 was completely hyper-phosphorylated in mitotic cells. S78A mutant was largely phosphorylated in mitotic cells similar to WT TREX1, but there is a detectable residual amount of TREX1 remained unphosphorylated. S261A mutant was largely unphosphorylated in mitotic cells, and S78A/S261A double mutant completely abolished TREX1 mitotic phosphorylation (Figure 2B). In contrast, S166A/S167A double mutant retained its mitotic phosphorylation but lost the double band in both interphase and mitotic cells (Figure 2C). All other mutants exhibited the double band in Phos tag containing SDS-PAGE. Therefore, the phosphorylation of S166/S167 is solely responsible for the appearance of the double band and is cell cycle independent. To further determine whether CDK1 directly phosphorylates TREX1 at S78 and S261, we performed in vitro kinase assay using recombinant CDK1 and immunoprecipitated WT or mutant TREX1 (V5 tagged). We found that WT TREX1 and S166A/S167A mutant were successfully phosphorylated by CDK1 in vitro, whereas S78A/S261A mutant was not phosphorylated (Figure 2D). Two additional triple and quadruple serine mutants (S78A/S166A/S261A and S78A/S166A/S167A/S261A) were also not phosphorylated (Figure 2D). Together, we conclude that mitotic phosphorylation of TREX1 occurs on S78 and S261 with S261 being the major phosphorylation site, and the mitotic phosphorylation is mediated by CDK1.

We also found that S78, S167, and S261 are conserved in various mammalian TREX1 homologs (Figure 2E). TREX1 DNase domain is highly conserved, but most of the linker region is not. Intriguingly, the CDK1 phosphorylation site at S261 falls in a motif with considerably higher conservation comparing to the rest of the linker region (Figure 2F). Together, we concluded that TREX1 is phosphorylated predominantly at S261 in mitotic cells, an evolutionarily conserved feature associated with the C terminus.

### TREX1 Is DNase Active during Mitosis, and the DNase Activity Is Not Affected by Phosphorylation

**Figure 3:**
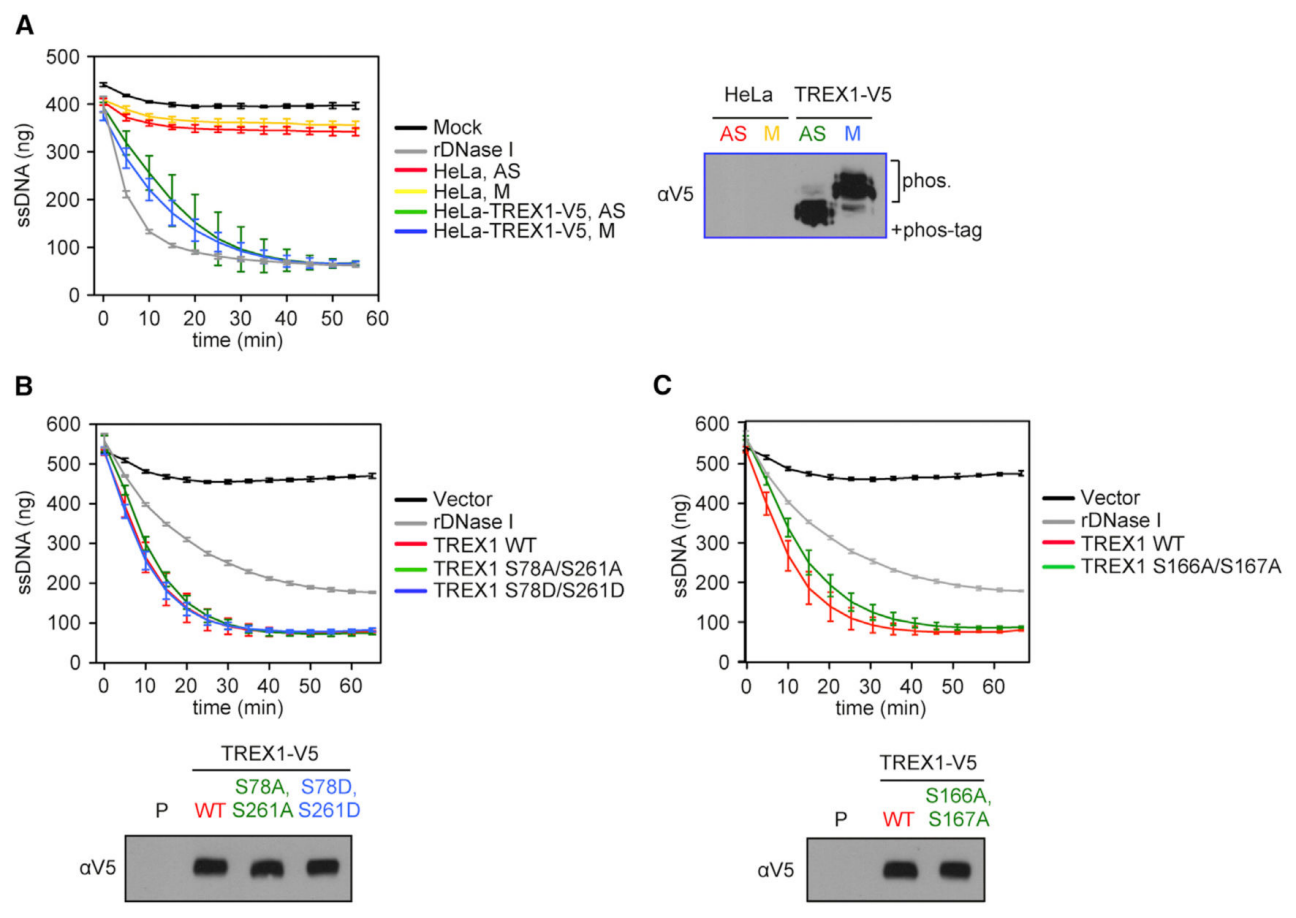
TREX1 DNase Activity Is Not Affected by Phosphorylation (A) TREX1 DNase activity was measured by a real-time fluorescent assay using a 30-nt-long ssDNA as a substrate. HeLa cells and HeLa cells stably expressing TREX1-V5 were grown asynchronously (AS) or arrested in mitosis with nocodazole (M). Cell extracts were immunoprecipitated with anti-V5 agarose beads. After the assay is completed, aliquots of the reaction mixtures were examined by immunoblot of Phos-Tag PAGE to confirm status of TREX1 phosphorylation (right). (B) TREX1-V5 WT, S78A/261A, and S78D/261D mutants were expressed in 293T cells and immunoprecipitated using anti-V5 agarose beads. DNase activity was measured as in (A). After the assay ended, aliquots of the reaction mixtures were examined by immunoblots with regular SDS-PAGE (bottom). (C) TREX1-V5 WT and a S166A/167A mutant were expressed in 293T cells and immunoprecipitated using anti-V5 agarose beads. DNase activity was measured as in (A). After the assay ended, aliquots of the reaction mixtures were examined by immunoblots with regular SDS-PAGE (bottom). Data are representative of at least three independent experiments. Error bars, SD.

We next determined whether TREX1 phosphorylation plays a role in regulating its DNase activity. First, we directly compared TREX1 DNase activity between interphase and mitosis, where TREX1 is found in its unphosphorylated and phosphorylated form, respectively. We established a stable line of HeLa cells expressing V5-tagged TREX1. We grew these cells asynchronously or arrested them in mitosis and immunoprecipitated TREX1-V5 in presence of phosphatase inhibitors. TREX1 DNase activity was measured using a previously established real-time fluoro-metric assay with 30-nt-long single-stranded DNA (ssDNA) as a substrate (Gehrke et al., 2013). We found no difference in DNase activities between the unphopshorylated and phosphorylated form of TREX1 (Figure 3A), suggesting that TREX1 is DNase active during mitosis. Next, we compared DNase activity of WT TREX1, phosphomimetic mutant S78D/S261D, and phosphor-deficient S78A/S261A and S166A/S167A mutants. All TREX1 double and quadruple serine mutants exhibited DNase activity indistinguishable from WT (Figures 3B, 3C, and S4). We also found that all serine mutants localize properly to the ER as WT TREX1 (Figure S5). Collectively, these experiments suggest that TREX1 is DNase active during mitosis, and DNase activity or ER localization is not affected by phosphorylation.

### Mitotic Phosphorylation Modulates a Transcriptome Signature Enriched in Glycol Genes

To explore the biological function of TREX1 mitotic phosphorylation, we stably expressed WT TREX1 (WT), S78A/S261A (S2A), or S78D/S261D (S2D) in *Trex1*^−/−^ MEFs using a retroviral system (Hasan et al., 2015). We then performed transcriptome analysis by RNA sequencing (RNA-seq). *Trex1*^−/−^ MEFs display elevated expression of IFN-stimulated genes (ISGs) due to innate immune activation, and re-expressing WT TREX1 suppresses the ISG signature (Hasan et al., 2013). We analyzed a panel of 35 ISGs that are elevated in *Trex1*^−/−^ MEFs compared to WT MEFs and found that both S2A and S2D mutant suppressed the expression of these ISGs in *Trex1*^−/−^ cells similar to WT TREX1 (Figures S6A and S6C and Table S1). We next examined genes that are differentially expressed in S2A and S2D compared to WT TREX1 rescue cells. We found 121 genes that are elevated by 1.5-fold or more in S2A or S2D or both comparing to WT TREX1 rescue cells. Gene oncology analysis did not yield any biological pathways that are significantly enriched in these genes. However, protein sequence feature analysis revealed significant enrichment of glycoproteins (35 genes, p = 8.1E-6), disulfide bond (32 genes, p = 2.4E-6), signal peptide (42 genes, 3.4E-7), and secreted proteins (31 genes, p = 5.6E-12), with largely overlapping genes in these categories (Figure S7). The expression of the 35 genes encoding glycoproteins (glycol genes) were significantly reduced in knockout (KO)/TREX1 cells compared to KO alone, and their expression in KO/S2A and KO/S2D were increased compared to KO/TREX1 cells (Figures S6B and S6D). Many of these glycol genes are also elevated in *Trex1*^−/−^ MEFs comparing to WT (Figure S6E). Because they are not associated with a particular network or pathway in contrast to the overwhelming evidence of elevated ISGs expression in *Trex1*^−/−^ cells, these glycol genes were not identified previously. Together, our data suggest that mitotic phosphorylation alters expression of a group of glycol genes, indicating potential dysregulation of OST activity and protein glycosylation.

### Mitotic Phosphorylation Disrupts TREX1 Interaction with the OST Complex

**Figure 4:**
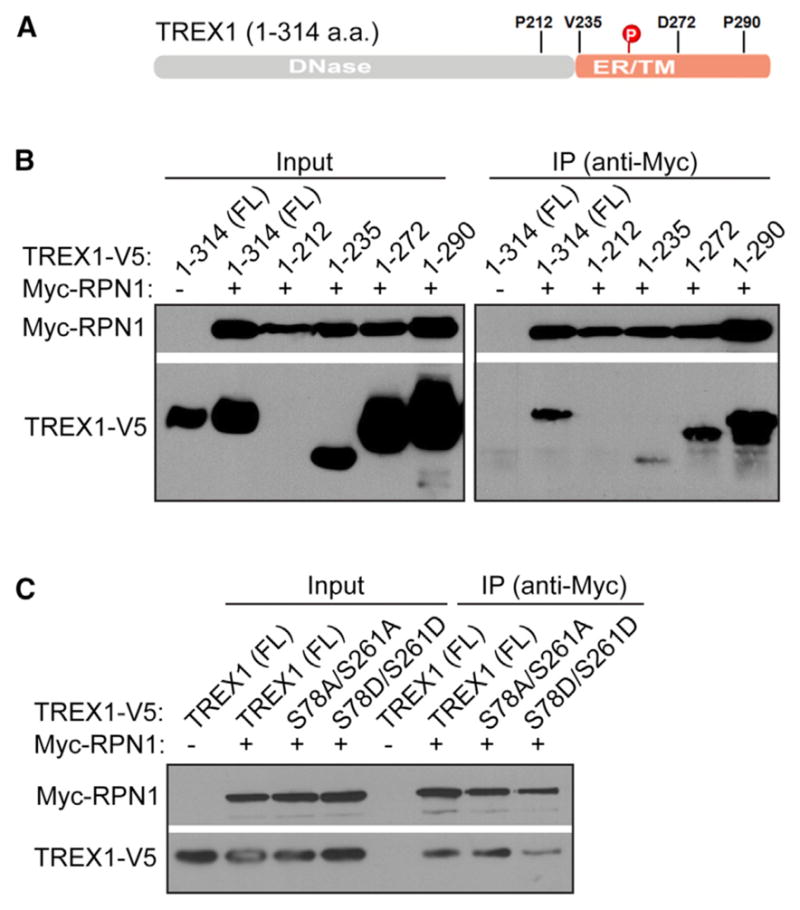
Mitotic Phosphorylation Disrupts TREX1 Interaction with the OST Complex (A) A schematic diagram of TREX1. Mitotic phosphorylation site S261 and truncation points used in (B) are shown. (B) Mapping TREX1:RPN1 interaction domain. V5-tagged WT TREX1 or C-terminal truncations and Myc-RPN1 were co-expressed in 293T cells. IP was performed with anti-Myc antibody. (C) Phosphomimetic mutation of S78/S261 disrupts RPN1 interaction. V5-tagged WT or mutant TREX1 and Myc-RPN1 were co-expressed in 293T cells. IP was performed with anti-Myc antibody. Data are representative of at least two independent experiments.

We next tried to determine whether mitotic phosphorylation alters TREX1 interaction with the OST complex. We showed previously that TREX1 C terminus harbors an DNase-independent function that regulates OST activity through interaction with its subunits (Hasan et al., 2015). To map the interaction domain, we generated several C-terminal truncations and examined their ability to interaction with OST subunit RPN1 (Figures 4A and 4B). We found that TREX1 WT and 1–290 co-immunoprecipitated with RPN1 to a similar level. Truncation 1-272 (mimic D272fs mutation associated with SLE) partially reduced TREX1:RPN1 interaction. Truncation 1-235 (mimic V235fs mutation associated with RVCL) nearly completely abolished TREX1:RPN1 interaction (Figure 4B). These interaction studies suggest that TREX1 interacts with RPN1 through a region between V235 and D272. Interestingly, this is the most evolutionarily conserved motif within the linker region (Figure 2F) that connects the N-terminal DNase domain and the C-terminal tail-anchor transmembrane domain.

Since the major mitotic phosphorylation site S261 falls exactly in this region, we next examined whether the two mitotic phosphorylation residues (S78 and S261) are required for TREX1:RPN1 interaction, or whether mitotic phosphorylation alters this interaction. S78A/S261A mutant did not affect TREX1:RPN1 interaction comparing to WT, suggesting these two residues are not required for interaction (Figure 4C). In contrast, S78D/S261D mutant (phosphomimetic) reduced TREX1:RPN1 interaction, suggesting mitotic phosphorylation disrupt TREX1 interaction with the OST complex (Figure 4C).

## DISCUSSION

*TREX1* mutations are associated with several autoimmune and inflammatory diseases. The different clinical etiologies of these diseases and their distribution into N or C terminus of the protein suggested at least two distinct functions of TREX1 (Hasan et al., 2015). The DNase domain located at the N terminus is critical for clearance of cytosolic DNA, and mutations disrupting DNase activity lead to activation of the innate immune cGAS-STING signaling pathway and elevated IFN and ISG signatures (Gao et al., 2015; Stetson et al., 2008). The C terminus of TREX1 is critical for ER localization, and we recently showed that it also regulates the activity of the OST complex involved in N-glycosylation (Hasan et al., 2015). Clusters of frameshift mutations truncating the C terminus lead to defects in glycans and potentially N-glycosylation without affecting the DNase activity (Hasan et al., 2015).

Here, we report the phosphorylation regulation of TREX1. We found that TREX1 is predominately phosphorylated at the S261 residue during mitosis by cyclin B/CDK1 and that TREX1 is dephosphorylated quickly upon mitotic exit likely by PP1/PP2 type serine/threonine phosphatase. This is distinct from cell-cycle-mediated phosphorylation of a related protein SAMHD1, which is phosphorylated in S phase by cyclin A/CDK2 (White et al., 2013). We are surprised to find that TREX1, which is locates on the surface of the ER with the DNase domain facing the cytosol (Wolf et al., 2016), is fully DNase active during mitosis, when it could gain access to nuclear genomic DNA. During cytotoxic T cell and Granzyme-mediated cell death, TREX1 translocates to the nucleus with the SET complex (containing other nuclease such as APEX1 and NM23H1) and causes DNA damage with the help of APEX1 and NM23H1 (Chowdhury and Lieberman, 2008). Our findings here demonstrate that TREX1 does not pose as a threat for genomic DNA damage during mitosis when the nuclear membrane breaks down, possibly due to the lack of other nucleases needed to nick double-stranded DNA (dsDNA) first or inaccessibility to compacted genomic DNA.

The major mitotic phosphorylation site S261 locates in a conserved motif within the linker region that connects the DNase domain and the tail-anchor transmembrane helix. The linker region as a whole is less conserved compared to the DNase domain, but interestingly all mammalian homologs contain a CDK1 consensus site in this area, indicating an evolutionary conserved function might be associated with this post-translational modification (PTM). We mapped the RPN1 interaction domain to this conserved motif within the linker region and demonstrated that phosphomimetic mutations of the mitotic phosphorylation sites S78 and S261 substantially disrupted TREX1:RPN1 interaction. We also found that mutations that disrupt the two mitotic phosphorylation sites alter the expression of a group of glycol genes, likely a functional consequence of dysregulated OST activity.

Together, we envision a model where TREX1 C terminus is phosphorylated (at S261) during mitosis, and this phosphorylation event disrupts the TREX1:OST interaction. Given the importance of TREX1 to stabilize OST on the ER, any disruption to this interaction could potentially lead to defects in N-glycosylation and glycan catabolism. The only exception may be during mitosis, when the nuclear membrane breaks down and protein translation is on hold, thus diminishing the need for N-glycosylation or a functional OST complex. Indeed, when we extended this phosphorylation beyond mitosis by mutating serine to aspatic acid as phosphomimetics, we blocked TREX1:RPN1 interaction constitutively and caused elevated expression of several glycol genes. Curiously, serine to alanine mutations at mitotic phosphorylation sites did not block TREX1:RPN1 interaction, while they also caused elevated expression of several glycol genes, suggesting mitotic phosphorylation or temporary separation of TREX1 from the OST complex may also be essential for OST activity. Further investigation is needed to better define this temporal regulation of OST and N-glycosylation during mitosis, which may shed light on the biology of the OST complex as well as mechanisms of immune diseases associated with TREX1 C terminus.

## EXPERIMENTAL PROCEDURES

### Cells, Plasmids, and Antibodies

HeLa, 293T cells, MEFs, and RAW264.7 were maintained in DMEM with 10% (v/v) heat-inactivated fetal bovine serum (FBS), 2 mM L-glutamine, 10 mM HEPES, and 1 mM sodium pyruvate (complete DMEM) with the addition of 100 U/mL penicillin, 100 mg/mL streptomycin, and cultured at 37°C with 5% CO_2_. Jurkat T cells, CEM, SupT1, and THP-1 cells were maintained in RPMI medium with 10% FBS with the same antibiotics. *Trex1*^−^*^/^*^−^ MEFs reconstituted with WT and mutant TREX1 were established using a retroviral system as described (Hasan et al., 2015). Antibodies used in this study include anti-TREX1 (mouse, 1:200 dilution, sc-271870, Santa Cruz Biotechnology), anti-mTREX1 (mouse, 1:1,000 dilution, 611987, Becton Dickinson), anti-STING (rabbit, 1:500 dilution, D2P2F, Cell Signaling Technology), anti-Cyclin B1 (rabbit, 1:500 dilution, sc-752, Santa Cruz), anti-tubulin (mouse, 1:20,000 dilution, B-5-1-2, Sigma), anti-V5 (mouse, 1:5,000, R-960-25, Life Technologies), anti-Myc (mouse, 1:5,000 dilution, sc-40, Santa Cruz), anti-calreticulin (rabbit, 1:1,000 dilution, ab4-100, Abcam), anti-V5 agarose beads (goat, 2 μg per immunoprecipitation [IP], S190-119, Bethyl Laboratories), and anti-Myc (rabbit, 2 μg per IP, sc-789, Santa Cruz).

### Flow Cytometry and Fluorescence Microscopy

For FACS analysis of the cell cycle, cells resuspended in PBS were fixed in cold 70% ethanol and washed once in PBS. After the PBS wash, cells were re-suspended in 500 μL of FxCycle PI/RNase Staining Solution (Thermo Fisher). Samples were processed using a FACSCalibur flow cytometer (BD Biosciences). Data were analyzed using the FlowJo software. For microscopy, HeLa cells grown on coverslips were transfected with either WT or mutant variants TREX1-V5 plasmids. 24 hr after transfection, cells were subsequently fixed in 4% paraformaldehyde and permeabilized in a 0.25% Triton X solution. Cells were later stained with anti-V5 and anti-calreticulin and Alexa Fluor 488 and 546 coupled secondary antibodies (Invitrogen) respectively. Coverslips were mounted in Vectashield (Vector Laboratories) mounting solution with DAPI and imaged using a Zeiss Imager M2 fluorescence microscope with AxioVision software.

### TREX1 In Vitro Phosphorylation and DNase Activity Assay

TREX1 exonuclease activity was assayed as described in Hasan et al. (2015). Briefly, approximately 1 × 10^6^ 293T were transfected with either WT or mutant TREX1-V5 plasmids. Cells were lysed in IP buffer (20 mM Tris-HCl [pH 7.4], 150 mM NaCl, 0.5% NP-40, and 1× protease inhibitor), and the post-nuclear supernatant was isolated by centrifugation at 20,000 × *g* and mixed with anti-V5 agarose beads (Bethyl Labs). Upon overnight incubation at 4°C, the beads were subsequently washed twice with IP buffer and twice with low-salt IP buffer (50 mM NaCl). Washed beads were resuspended in 50 μL of DNase buffer (20 mM Tris-HCl [pH 7.4], 5 mM MgCl_2_, 2 mM DTT, 100 mg/mL BSA, and 0.5% NP-40). Bead-bound TREX1 was subject to either in vitro phosphorylation or DNase assay. For TREX1 in vitro phosphorylation, beads bound TREX1 was incubated with 20U of recombinant CDK1 (NEB) following the manufacturer’s suggestion. Samples were later analyzed by Phos-tag immunoblotting. To assess TREX1 DNase function, samples were divided into three equal volumes and mixed with 90 μL of pre-warmed to 37°C DNase reaction buffer (20 mM Tris-HCl [pH 7.4], 5 mM MgCl_2_, 2 mM DTT, 100 mg/mL BSA, 1/1,200 SYBR Green, and 5 ng/μl or 10 ng/μl ssDNA) followed by real-time quantification of the DNA/SYBR complex using a SynergyHT microplate reader (Biotek).

### Co-immunoprecipitation

Approximately 2 × 10^6^ 293T cells were transfected with WT, mutant variants, or truncated variants TREX1-V5 and Myc-RPN1 plasmids. Cells were collected and lysed as described above. The supernatant was subsequently mixed with anti-Myc and Dynabeads Preotein G (Life Technologies) and incubated overnight at 4°C. Next, samples were washed once with IP buffer, twice with high-salt IP buffer (500 mM NaCl) and once with a low-salt IP buffer (50 mM NaCl). Immunocomplexes were eluted with 3× sample buffer and boiling. Samples were subsequently analyzed by immunoblotting.

### Phos-Tag SDS-PAGE

Mitosis synchronized or non-synchronized cells were collected and lysed in radioimmunoprecipitation assay (RIPA) buffer with 1× protease inhibitors and phosphatase inhibitors (25 mM NaF and 1 mM Na_3_VO_4_). The post-nuclear supernatant protein content was determined using Pierce BCA protein Assay (Thermo). Samples were either treated with Lambda Protein Phosphatase (NEB) or loaded directly into a 10% polyacrylamide Phos tag gel (50 μM Phos-Tag [Wako] and 100 μM MnCl_2_). Prior to electroblotting, gels were soaked in transfer buffer with 1 mM EDTA for 10 min. Samples were subsequently analyzed by immunoblotting.

### Cell-Cycle Synchronizations

For G1/S synchronization (double-thymidine block), HeLa cells were plated at 25%–30% confluency and treated with 2 nM thymidine for 18 hr. Then, cells were washed two times with warm PBS and grown in fresh medium for 9 hr. Then, cells were treated again with 2 mM thymidine for 15 hr to arrest at G1/S phase. In selective experiments, cells were released into S phase by washing two times with warm PBS and adding fresh conditioned medium. For M phase synchronization (thymidine-nocodazole block), cells were plated at 40% confluency and treated with with 2 nM thymidine for 24 hr. Then, cells were washed two times with warm PBS and grown in fresh medium for 3 hr. Cells were then treated with 100 ng/mL Nocodazole for 12 hr. Dishes were knocked with knuckles to dislodge mitotic cells from the plate. These round cells are arrested in mitosis. To release these cells into G1, we removed nocodazole by washing two times with warm PBS and adding fresh medium.

### Statistical Methods

Data are presented as the mean ± SEM. GraphPad Prism 6 was used for statistical analysis. Statistical tests performed were indicated in the figure legends. *p < 0.05, **p < 0.01, ***p < 0.001, and ****p < 0.0001.

## Supplementary Material

Supplemental Material

## Highlights

- During mitosis, TREX1 is predominately phosphorylated at Serine-261 by Cyclin B/CDK1
- Mitotic phosphorylation does not affect TREX1 DNase activity
- Mitotic phosphorylation disrupts TREX1 interaction with the OST subunit RPN1
- Disrupting TREX1 phosphorylation at Serine-261 elevates a glycol-gene signature
