# Supplementary material for: Mitotic Phosphorylation of TREX1 C Terminus Disrupts TREX1 Regulation of the Oligosaccharyltransferase Complex
Source: Cell Rep. Author manuscript; Available in PMC 2017 Nov 27. (PMC5703050; doi:10.1016/j.celrep.2017.02.051)
Supplement: Supplemental Material [file NIHMS891741-supplement-Supplemental_Material.pdf]

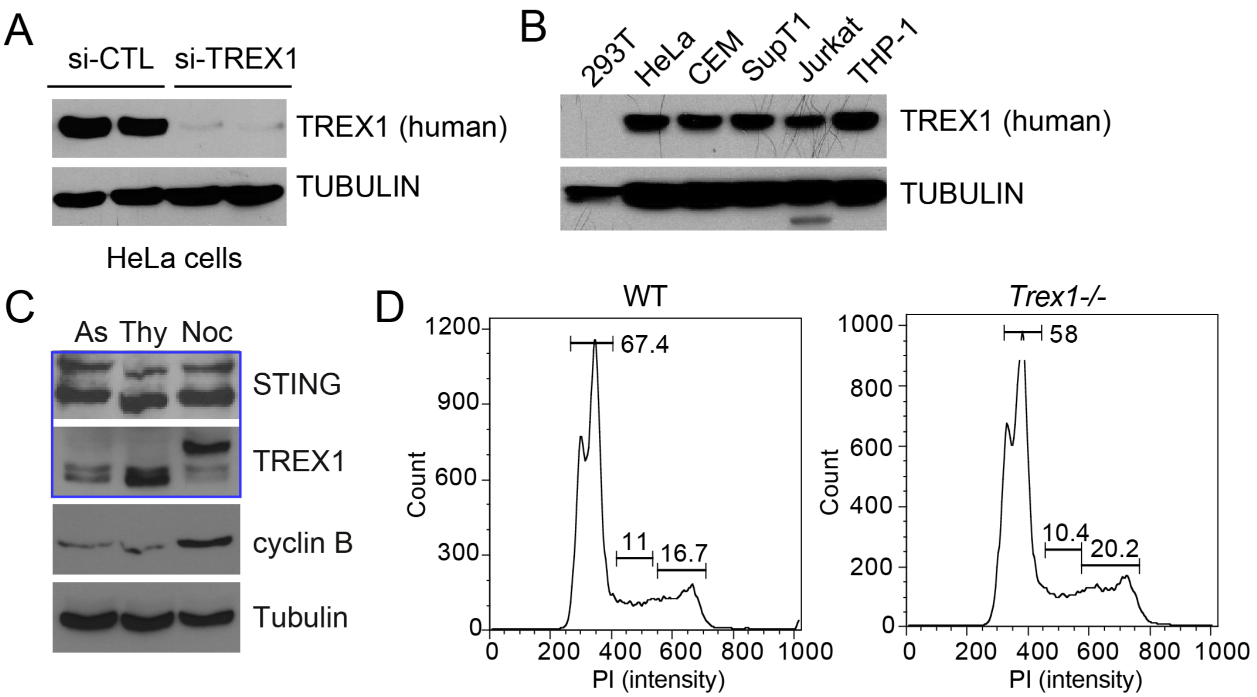

Figure S1. TREX1 antibody specificity, STING phosphorylation and cell cycle. Related to **Figure 1**. (A) siRNA knockdown of endogenous TREX1 in HeLa cells. HeLa cells were transfected with control or TREX1 specific siRNA for 48 hours. Endogenous TREX1 was analyzed by Western blot. (B) Endogenous TREX1 expression in various human cell lines (indicated on top). Note that 293T cells do not express detectable endogenous TREX1, which was use for in vitro DNase activity assay. (C) STING phosphorylation is not regulated by the cell cycle. HeLa cells were grown asynchronously (As), arrested with thymidine in interphase (Thy), or arrested in mitosis with nocodazole (Noc) as in Figure 1A. STING and TREX1 phosphorylation was assayed using immunoblot of Phos-Tag PAGE. Cyclin B and Tubulin (loading control) was assayed using immunoblot of regular SDS-PAGE. (D) TREX1 does not regulate cell cycle. Wild type and *Trex1*-/- MEFs were stained with propidium iodide (PI) and analyzed by FACS. No appreciable difference in cell cycle was observed. Data are representative of at least three independent experiments.

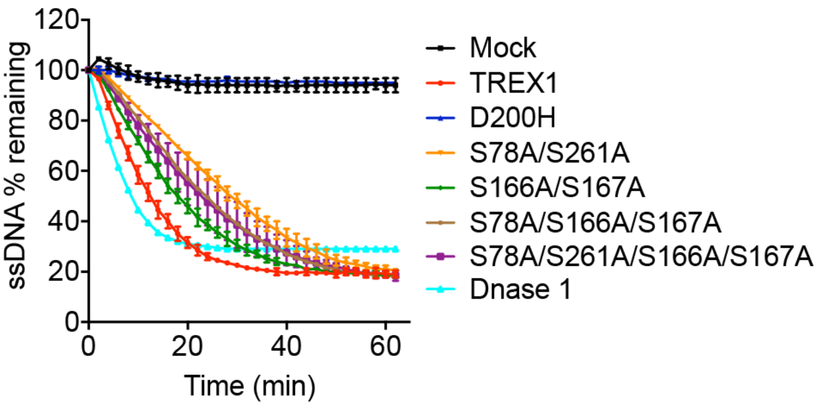

Figure S2. TREX1 serine mutants remain DNase active. Related to **Figure 3.** V5-tagged TREX1 serine mutants were expressed in 293T cells, immunoprecipitated with V5 beads, and subjected to in vitro DNase assay as in **Figure 3**. All serine mutants remain DNase active similar to wild type TREX1. D200H is known to be DNase-dead.

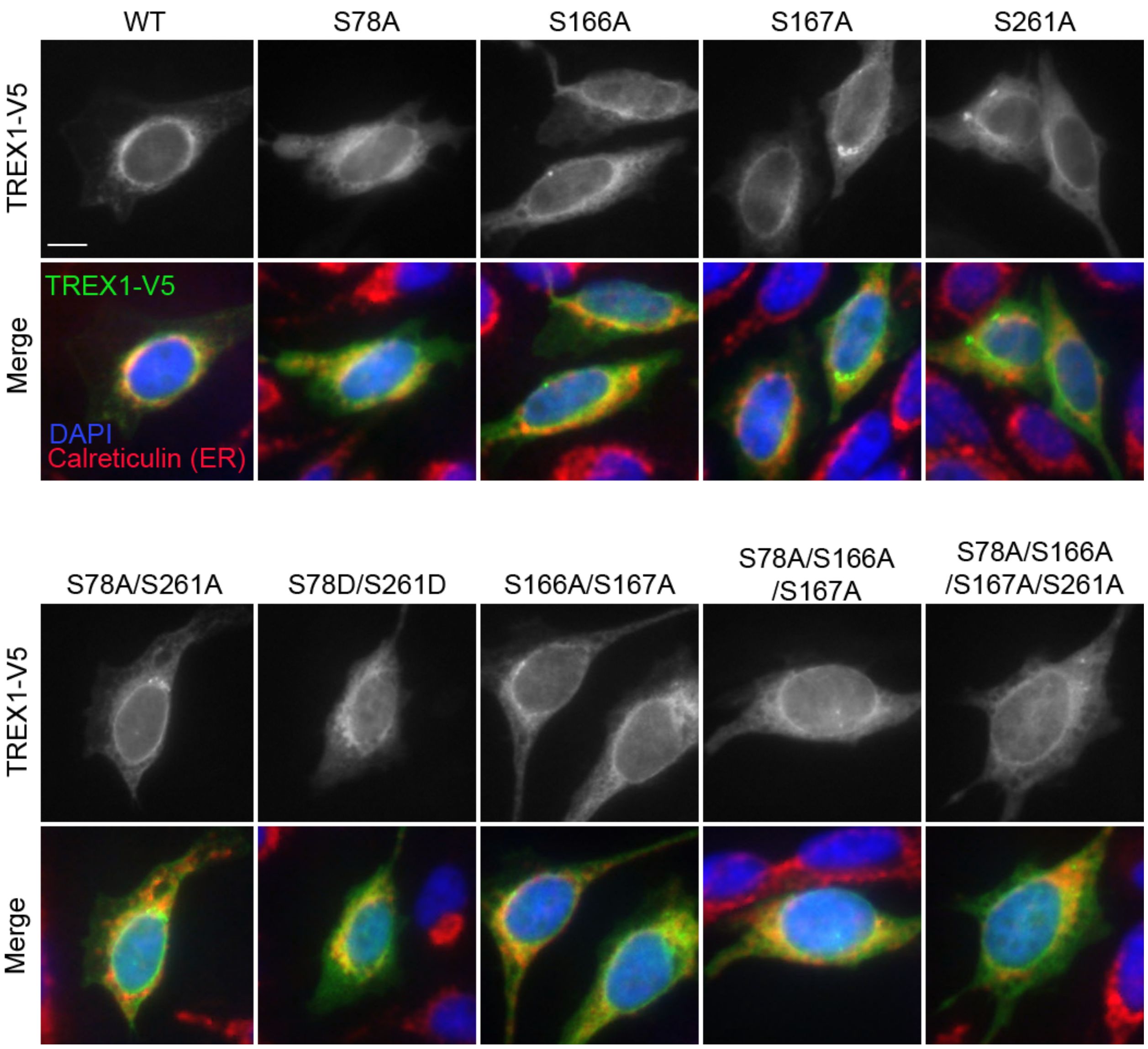

Figure S3. TREX1 serine mutants localize to the ER properly. Related to **Figure 3.** HeLa cells transiently transfected with V5-tagged wild type or mutant TREX1 were stained with anti-V5 (green) and anti-Calreticulin (red, an ER marker). Nucleus was stained with DAPI (blue). Top panels show the V5 channel only (in monochrome) and bottom panels show the merge of all three channels (in color). Scale bar, 10 μm.

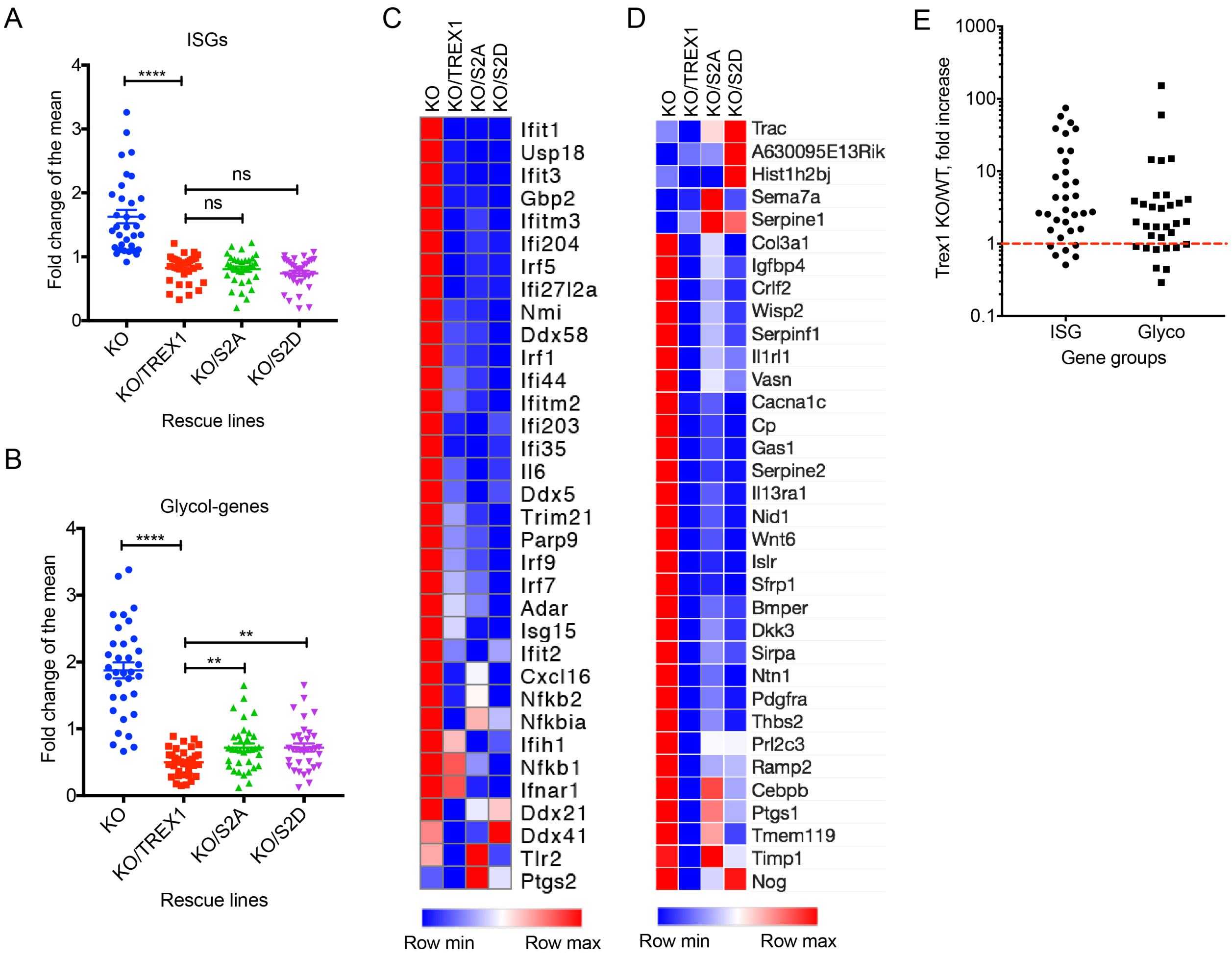

F

Rank	Term	Count	%	P-Value	Genes
1	Secreted	31	25.6	5.57E-12	CXCL1, NOG, CRLF2, EDN1, COL3A1, TIMP3, TIMP1, PRL2C3, ISLR, WISP2, SERPINE2, CTGF, SBSN, SERPINE1, WNT6, PRL2C2, IL1RL1, 2300009A05RIK, NID1, IGF2, COL5A3, NTN1, A630095E13RIK, DKK3, BMPER, SFRP1, SERPINF1, PPBP, NPPB, CP, IGFBP4
2	Signal	42	34.7	3.39E-07	CXCL1, NOG, LRRC32, CRLF2, PTGS1, COL3A1, EDN1, TIMP3, TIMP1, PRL2C3, ISLR, WISP2, SERPINE2, CTGF, SEMA7A, SBSN, SERPINE1, IL13RA1, WNT6, THBS2, PRL2C2, VASN, RAMP2, IL1RL1, 2300009A05RIK, NID1, IGF2, GAS1, COL5A3, NTN1, SIRPA, A630095E13RIK, DKK3, BMPER, SFRP1, SERPINF1, PPBP, PDGFRA, NPPB, CP, TMEM119, IGFBP4
3	Disulfide bond	32	26.4	2.40E-06	CXCL1, S100A3, NOG, CRLF2, PTGS1, EDN1, COL3A1, TIMP3, TIMP1, PRL2C3, ISLR, CTGF, SEMA7A, WNT6, IL13RA1, THBS2, PRL2C2, VASN, RAMP2, IL1RL1, NID1, IGF2, NTN1, SIRPA, A630095E13RIK, DKK3, SFRP1, PDGFRA, NPPB, CP, CACNA1C, IGFBP4
4	Glycoprotein	35	28.9	8.12E-06	NOG, CRLF2, PTGS1, COL3A1, TIMP1, PRL2C3, ISLR, WISP2, TRAC, SERPINE2, SEMA7A, HIST1H2BJ, SERPINE1, WNT6, IL13RA1, THBS2, PRL2C2, VASN, RAMP2, CEBPB, IL1RL1, NID1, GAS1, NTN1, SIRPA, A630095E13RIK, DKK3, BMPER, SFRP1, SERPINF1, PDGFRA, CP, TMEM119, CACNA1C, IGFBP4

Figure S4. Mitotic phosphorylation modulates a transcriptome signature enriched in glycol-genes. Related to **Figure 4.**

(A, B) Group presentation showing expression of 35 IFN stimulated genes (ISGs) (A) or 35 glycol-genes (B) in *Trex1*-/- MEFs (KO) reconstituted with wild type TREX1 (KO/TREX1), S78A/S261A mutant (KO/S2A) or S78D/S261D (KO/S2D) mutant. Each dot represents a different gene. Expression values of each gene in four rescue cell lines were presented as fold change of the mean. (C, D) Heatmap presentation showing expression of ISGs (C) and glycol-genes (D) using the same RNAseq data set as in A and B. Heatmaps were generated using Morpheus (Broad Institute) with each row individually normalized to show high in red and low in blue. (E) Expression of ISGs and glyco-genes in Trex1 KO MEFs compared to WT MEFs. Data in A-D are from one set of RNA-seq comparing four rescuing MEF cells. Data in E is from a previous published RNA-seq comparing WT and Trex1 KO MEFs (Hasan et al., 2013). **P < 0.01, ****P < 0.001. ns = not significant. Unpaired t-test. Error bars, SEM. (F) Glycol-genes enriched in KO/S2A and KO/S2D rescue cells. DAVID analysis (https://david.ncifcrf.gov) of 121 genes up-regulated in KO/S2A or KO/S2D compared KO/TREX1. Most enriched sequence features and associated genes are shown. RNA-seq dataset in **Table S1**.

Table S1. RNA-seq analysis of Trex1-KO, KO/TREX1-WT, KO/S2A and KO/S2D MEFs. Related to **Figure 4.**
